# A Case of Treatment‐Refractory Tumefactive Multiple Sclerosis Mimicking Brain Abscess

**DOI:** 10.1155/crnm/1858367

**Published:** 2026-10-30

**Authors:** Pooja Patel, Henrik Ehrhardt, Daniel Augh, Kant Matsuda, Kanika Sharma

**Affiliations:** ^1^ Department of Neurology Rutgers Robert Wood Johnson Medical School New Brunswick New Jersey USA rutgers.edu; ^2^ Department of Pathology Rutgers Robert Wood Johnson University Hospital New Brunswick New Jersey USA rwjuh.edu

## Abstract

A 47‐year‐old female presented with one week of left facial numbness following recent dental extractions. Initial brain magnetic resonance imaging (MRI) with and without contrast revealed a contrast‐enhancing lesion in the right parieto‐occipital lobe with mild diffusion restriction and surrounding vasogenic edema, as well as smaller enhancing lesions in the right frontal and parietal lobes. Empiric intravenous (IV) antibiotic treatment was started due to a high concern for odontogenic brain abscesses. Despite antibiotic treatment, repeat brain MRI revealed new contrast‐enhancing lesions in the left pons and cerebellar peduncle, suggesting that infection was unlikely and antibiotics were discontinued. The differential diagnosis broadened to neoplastic, inflammatory, and demyelinating pathologies. A brain biopsy of the right occipital lobe lesion was pursued for a definitive diagnosis. Following the brain biopsy, the patient received five days of IV methylprednisolone 1 g daily, followed by a two‐week oral prednisone taper with clinical improvement. Brain biopsy subsequently demonstrated macrophages, reactive lymphocytosis, and astrogliosis with loss of myelin, confirming the diagnosis of tumefactive multiple sclerosis (TMS). Over the subsequent two years, she experienced recurrent flares of TMS with new demyelinating lesions despite disease‐modifying therapy (DMT) with ocrelizumab, resulting in progressive disability accrual. Upon transition to natalizumab, disease remission was achieved. This case highlights the diagnostic challenges of TMS as it may radiographically resemble odontogenic brain abscesses, particularly in the setting of recent dental infections. Brain biopsy may be required when neuroimaging and cerebrospinal fluid findings are inconclusive. This case also suggests that natalizumab may be considered in selected treatment‐refractory cases, although further evidence is needed.

## 1. Introduction

Tumefactive multiple sclerosis (TMS) is a rare variant of multiple sclerosis. TMS causes demyelinating brain lesions that appear as large, tumor‐like masses with contrast enhancement and vasogenic edema, which may radiographically resemble infectious, neoplastic, and inflammatory pathologies, thus posing diagnostic challenges [1, 2]. In cases of diagnostic uncertainty, a brain biopsy may be necessary for a definitive diagnosis. Histopathological features of TMS include hypercellular lesions with abundant macrophages, myelin loss, reactive astrogliosis, and relative axonal preservation [2].

Given that TMS can present with highly active disease, prompt recognition of an acute flare and timely treatment with immunotherapy are crucial for disease control. Acute treatment of flares includes high‐dose intravenous (IV) corticosteroids or plasma exchange (PLEX) for refractory cases [3]. Maintenance treatment with a disease‐modifying therapy (DMT) is recommended to reduce the risk of recurrent relapses and disability accrual. In the current literature, evidence‐based guidance for optimal DMT selection in TMS is limited to case reports and case series.

We report an aggressive, treatment‐refractory case of TMS in a middle‐aged female who initially presented with multiple enhancing lesions in the setting of extensive dental extractions, which raised strong concern for odontogenic brain abscesses. A brain biopsy ultimately confirmed the diagnosis of TMS. Despite treatment with ocrelizumab, the patient experienced recurrent, disabling TMS flares and required escalation to a high‐efficacy DMT, natalizumab. This case highlights the diagnostic complexity of TMS, the substantial disease burden associated with recurrent relapses, and the therapeutic challenges despite escalation of maintenance immunotherapy.

## 2. Case Presentation

A 47‐year‐old female with a history of recent dental extractions of sixteen decayed teeth presented with one week of left facial numbness and swelling. She noted that the onset of her left facial numbness preceded her dental extractions by five days. Vital signs included a temperature of 98.2°F, blood pressure of 157/78 mmHg, heart rate of 78 bpm, and respiratory rate of 18 breaths/minute. On examination, she was in no acute distress and appeared nontoxic. Oral examination revealed left mandibular swelling, abnormal dentition with dental tenderness, and gingival swelling. Neurological examination was notable for reduced sensation to light touch in the left V1‐V2 region. Computed tomography (CT) of the head without contrast demonstrated hypodensities in the right parietal and occipital lobes. Brain magnetic resonance imaging (MRI) with and without contrast demonstrated an 11 × 18 mm enhancing lesion in the right parieto‐occipital region with mild diffusion restriction and vasogenic edema, as well as small enhancing lesions in the right frontal and parietal lobes (Figures 1A–D). There were no paramagnetic rim lesions or central vein signs. Given the patient’s history of extensive dental caries, there was a high concern for odontogenic brain abscesses due to spread from her dental infections, for which she received empiric antibiotic treatment with IV vancomycin, ceftriaxone, and metronidazole. Infectious workup was unrevealing, with negative blood cultures, serum QuantiFERON‐TB Gold test, nonreactive rapid plasma reagin (RPR), HIV screen, and cryptococcal antigen. CT of the chest, abdomen, and pelvis showed no signs of occult malignancy, and the patient was up to date with her cancer screenings. A timeline of the patient’s clinical course is depicted in Table 1.

**FIGURE 1:**
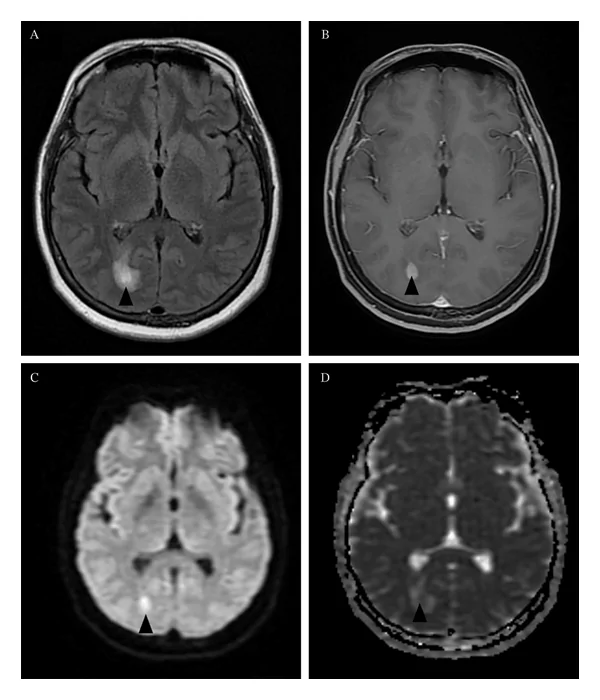
(A) MRI brain with and without contrast T2‐FLAIR sequence demonstrating a hyperintense lesion in the right parieto‐occipital lobe. (B) MRI brain with and without contrast T1‐postcontrast sequence demonstrating a contrast‐enhancing lesion in the right parieto‐occipital lobe. (C) MRI brain with and without contrast DWI sequence demonstrating a hyperintense lesion in the right parieto‐occipital lobe with mild diffusion restriction. (D) MRI brain with and without contrast ADC sequence demonstrating a hypointense lesion with low signal in the right parieto‐occipital lobe, consistent with mild diffusion restriction.

**TABLE 1:** Timeline of clinical course.

Timeline	Clinical course	Key findings	Treatment	Response
Week 0	Onset of left facial numbness			
Week 1, Day 1	Outpatient dental extractions followed by initial hospitalization	Brain MRI: enhancing lesion in the right parieto‐occipital region and small enhancing lesions in right frontal and parietal lobes	Initiation of empiric antibiotics	Persistent left facial numbness
Week 1, Day 6	Persistent left facial numbness	Brain MRI: new enhancing left pontine and left cerebellar peduncle lesions. LP #1: mild lymphocytic pleocytosis, absent oligoclonal bands	Discontinuation of antibiotics	Persistent left facial numbness
Week 2	Improvement of left facial numbness	Brain MRI: increased size of the right occipital lobe and left cerebellar peduncle lesions. LP #2: mild lymphocytic pleocytosis, absent oligoclonal bands		Improvement of left facial numbness
Week 3	Brain biopsy, followed by initiation of empiric corticosteroid treatment		IV methylprednisolone 1 g daily × 5 days transitioned to oral steroid taper	Improvement of left facial numbness
Month 2	Brain biopsy confirmation of tumefactive multiple sclerosis			
Month 3	Relapse #1: headache, nausea, vomiting, and generalized weakness	Brain MRI: new enhancing lesions involving the splenium of the right corpus callosum, bilateral paracentral white matter, and the right superior cerebellar peduncle	IV methylprednisolone 1 g × 8 days. First dose of ocrelizumab administered inpatient	Improvement in symptoms
Month 5	Relapse #2: Right eye vision loss	Brain MRI: new enhancing lesions involving the left temporo–parietal‐occipital region and the splenium of the left corpus callosum, with markedly worsened vasogenic edema in the right cerebral hemisphere	IV methylprednisolone 1 g daily × 5 days, followed by alternating PLEX × 5 days with IV methylprednisolone 500 mg daily × 5 days on non‐PLEX days	Mild improvement in right eye vision
Month 9	Relapse #3: bilateral vision loss	Brain MRI: increased size of the left temporo–parietal‐occipital enhancing lesions with worsened edema	Alternating PLEX × 5 days with IV methylprednisolone 1 g daily × 5 days	Improvement in vision
Month 11	Continuation of ocrelizumab as DMT		Second dose of ocrelizumab	
Year 2, Month 1	Relapse #4: left upper and lower extremity weakness	Brain MRI: new enhancing lesion of the right centrum semiovale and corona radiata	IV methylprednisolone 1 g × 5 days followed by alternating PLEX × 5 days with IV methylprednisolone 500 mg daily × 5 days on non‐PLEX days	Clinical and radiographic improvement
Year 2, Month 2	Initiation of natalizumab as DMT		First dose of natalizumab	
Year 2, Month 3	Relapse #5: worsened left upper and lower extremity weakness	Brain MRI: increased size and nodular enhancement of the prior lesions in the right centrum semiovale and corona radiata	IV methylprednisolone 1 g daily × 3 days followed by PLEX × 5 days	
Remission duration: 1 year	Remission of TMS		Continuation of natalizumab as DMT	Clinical and radiographic improvement

Repeat brain MRI with and without contrast was performed five days after the initial brain MRI and demonstrated new enhancing lesions in the left pons involving the left trigeminal nerve and the left cerebellar peduncle (Figure 2A‐B). MRI of the cervical, thoracic, and lumbar spine with and without contrast demonstrated no spinal lesions. The patient reported that her left‐sided facial numbness was still present but had improved. Neurological examination demonstrated reduced sensation to light touch and temperature in the left V1‐V2 distribution but was otherwise unchanged from her initial evaluation. A lumbar puncture (LP) was performed and revealed cerebrospinal fluid (CSF) glucose 73 mg/dL (normal: 40–80 mg/dL), protein 30 mg/dL (normal: 15–45 mg/dL), cell count of 7 cells/μL (7 lymphocytes and 26 RBCs), absent oligoclonal bands, negative meningitis/encephalitis panel, negative cytology, and negative *Toxoplasma* polymerase chain reaction (PCR). Antibiotics were discontinued after a six‐day course due to low likelihood of infection and clinical stability. At this time, CNS malignancy, including CNS lymphoma, and demyelinating/inflammatory conditions were considered most likely.

**FIGURE 2:**
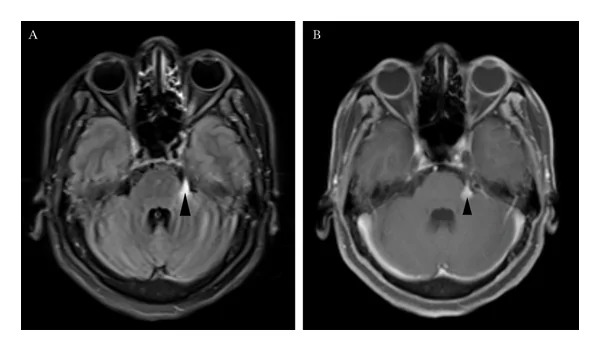
(A) MRI brain with and without contrast T2‐FLAIR sequence demonstrating a hyperintense lesion in the left pons involving the left trigeminal nerve. (B) MRI brain with and without contrast T1‐postcontrast sequence demonstrating an enhancing lesion in the left pons involving the left trigeminal nerve.

Brain MRI with and without contrast was repeated one week later and demonstrated increased size of the previously enhancing lesions in the right occipital lobe and left cerebellar peduncle. A LP was repeated one week following her initial LP and yielded CSF glucose 67, protein 75 mg/dL, cell count 10 cells/μL (8 lymphocytes, 2 monocytes, and 0 RBCs), absent oligoclonal bands, and negative cytology and cytometry. CNS lymphoma was felt to be less likely after two unrevealing LPs. Given worsened radiographic lesions and inconclusive CSF results, a brain biopsy was proposed for definitive diagnosis. The patient underwent a brain biopsy of her right occipital lobe lesion, which was complicated by a new left homonymous hemianopsia. While awaiting the results of her brain biopsy, she received treatment with five days of IV methylprednisolone 1 g daily, followed by a two‐week oral prednisone taper, for a suspected inflammatory or demyelinating pathology. Following her steroid course, she noted marked improvement in her left facial numbness. She was discharged home with the plan to follow up in the outpatient neuroimmunology clinic to discuss her remaining test results.

At an outpatient follow‐up, the remaining test results included negative serum myelin oligodendrocyte glycoprotein (MOG) antibody, Aquaporin‐4 antibody, and ACE, thereby suggesting that MOG antibody–associated disease (MOGAD), neuromyelitis optica spectrum disorder (NMOSD), and neurosarcoidosis were unlikely. Brain biopsy demonstrated a macrophage‐rich lesion with reactive lymphocytosis, astrogliosis, and loss of myelin on Luxol Fast Blue stain, confirming the diagnosis of TMS (Figures 3A–F).

**FIGURE 3:**
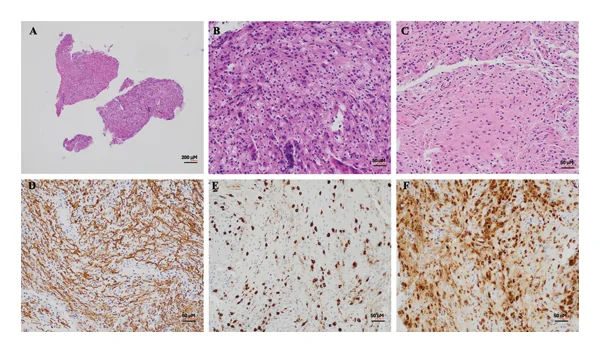
Brain biopsy of right occipital lobe. (A) Hematoxylin and eosin (H&E) demonstrating small fragments of biopsy tissue at low power magnification (20x). (B) H&E demonstrating mixed lymphohistiocytic infiltrate at high‐power magnification (200x). (C) Myelin stain with Luxol Fast Blue (LFB) demonstrating total loss of myelin. (D) Immunohistochemistry (IHC) demonstrating preservation of axons. (E) IHC for CD3 T‐cells. (F) IHC for CD68 macrophages.

Since then, the patient experienced many flares of TMS with the development of new demyelinating lesions in the bilateral supratentorial and infratentorial brain during each relapse. Table 1 details the clinical time course of the patient’s relapses and subsequent remission. During her flares, she received treatment with varying combinations of short courses of high‐dose IV methylprednisolone and PLEX with clinical and radiographic improvement. She was initially treated with ocrelizumab as a DMT. However, she continued to have subsequent flares with the development of new lesions and increased disability. During the first six months of treatment with ocrelizumab, she experienced two flares with significant neurological disability accrual. These relapses represented breakthrough inflammatory disease, which raised concern for a suboptimal response to ocrelizumab. She was transitioned to monthly infusions of natalizumab (300 mg every 28 days) with subsequent remission of her disease. One month prior to starting natalizumab, John Cunningham virus (JCV) PCR of whole blood and CSF were performed and resulted negative in both specimens. Thereafter, she has undergone monthly evaluations of serum JCV antibody index, which initially increased from 1.01 to a maximum of 2.74 but most recently decreased to 2.54. Given the rising JCV antibody index and the need to maintain clinical stability while continuing natalizumab therapy, the dosing interval was extended to every six weeks.

At her most recent clinic appointment, her visual acuity was OD 20/50, OS 20/50, and 20/70 OU, with left homonymous hemianopsia, reduced left facial sensation to light touch, and 4/5 strength in the left upper and lower extremities with intact reflexes. She has remained in remission for one year with monthly natalizumab infusions. Brain MRI, repeated one year after clinical remission, demonstrated resolution of the prior demyelinating lesions, with no evidence of active disease.

## 3. Discussion

TMS is a rare variant of multiple sclerosis with an estimated prevalence of 1–3 per 1000 cases of MS per year [4]. The radiographic appearance of tumefactive demyelinating lesions (TDLs) on brain MRI appears as tumor‐like lesions, typically ≥ 2 cm in diameter, with contrast enhancement and vasogenic edema [2]. TDLs demonstrate variable contrast enhancement patterns, either heterogeneous or homogeneous enhancement, as well as open or closed ring enhancement [2]. The most common locations of TDLs are the frontal, parietal, and temporal lobes [5]. Other regions vulnerable to tumefactive demyelination include the periventricular region, corpus callosum, brainstem, and spinal cord [2]. The clinical manifestations of TMS are dependent on the location and size of the lesions and may include headaches, encephalopathy, seizures and focal weakness [2]. Our patient had extensive, widespread demyelinating lesions affecting the bilateral hemispheres, multiple lobes, periventricular region, corpus callosum, and brainstem. Initially, this patient’s demyelinating lesions were smaller than 2 cm; however, subsequent relapses were characterized by TDLs exceeding 2 cm. The demyelinating lesions of TMS may radiographically resemble various infectious, neoplastic, and inflammatory space–occupying lesions, which may contribute to diagnostic delays, as observed in this case [1].

In this case, there was an initial high suspicion for pyogenic brain abscesses given the patient’s history of extensive dental caries requiring extractions. Oral bacteria can spread hematogenously from dental inflammation to the brain and cause life‐threatening brain abscesses due to transient bacteremia and reduced immunity [6]. Patients with odontogenic disease, including dental caries, periodontal abscesses, gingivitis, and periodontitis, are particularly at risk [7]. On brain MRI, brain abscesses demonstrate diffusion restriction and distinct contrast enhancement, including a hyperintense capsule and a hypointense necrotic core [8]. Our patient did not clinically improve despite antibiotic treatment and subsequently developed new brain lesions. Therefore, brain abscesses were felt to be unlikely, and antibiotics were discontinued. Following the exclusion of odontogenic brain abscess, neoplastic, inflammatory, and demyelinating conditions were further prioritized in the differential diagnosis.

Primary central nervous system lymphoma (PCNSL) is a rare type of lymphoma confined to the central nervous system that may involve the eyes, brain, leptomeninges, and spinal cord without evidence of systemic disease [9]. On brain MRI, PCNSL typically presents as a single, homogeneously enhancing intraparenchymal lesion with diffusion restriction located in the supratentorial region, most commonly in the cerebral hemispheres. Multiple high‐volume LPs are recommended to achieve high sensitivity from CSF cytology and cytometry collections [10]. Histopathological confirmation with a brain biopsy is the gold standard method to diagnose PCNSL [10]. In our patient, two LPs were performed with unremarkable CSF studies, lowering the likelihood of PCNSL. A brain biopsy was pursued for definitive diagnosis as the radiographic appearance of the lesions resembled PCNSL, as well as demyelinating and inflammatory conditions. In the evaluation of PCNSL, corticosteroids should be avoided prior to brain biopsy as they may cause partial radiographic recovery, described as “vanishing tumor,” leading to false‐negative or nondiagnostic biopsies [11]. In our patient, high‐dose IV methylprednisolone was initiated following her brain biopsy due to a high suspicion for a demyelinating or inflammatory condition.

As there was neuroimaging overlap with inflammatory and demyelinating neurological conditions, further evaluation was pursued. Demyelinating conditions, including TMS, NMOSD, and MOGAD, were considered, prompting additional serological testing. Oligoclonal bands were not found in the CSF, which would have suggested an immune‐mediated etiology, though their absence did not exclude one. NMOSD is a demyelinating disease caused by antibodies against Aquaporin‐4 and may cause optic neuritis, cerebral lesions, and longitudinally extensive transverse myelitis (LETM) [12]. On brain MRI, NMOSD can cause nonspecific heterogeneous brain lesions in the deep white matter, LETM, and severe, often bilateral optic neuritis [13]. MOGAD is a demyelinating disease characterized by antibodies against MOG and may also cause optic neuritis, cerebral lesions, and LETM [12]. In our patient, NMOSD and MOGAD were excluded based on negative serum Aquaporin‐4 and anti‐MOG antibody testing, respectively. Neurosarcoidosis is an inflammatory granulomatous disease, which may cause leptomeningeal and pachymeningeal enhancement, cranial neuropathies, hydrocephalus, and intraparenchymal brain and spinal cord lesions [14]. On brain MRI, neurosarcoidosis may present as small linear or nodular lesions located on the meninges or along perivascular routes [13]. Rarely, neurosarcoidosis may manifest as tumefactive brain lesions, reported to be greater than 1 cm with perilesional edema and mass effect, typically located in the supratentorial region and associated with leptomeningeal or pachymeningeal enhancement [15]. Neurosarcoidosis was considered less likely with negative serum ACE and the absence of other supportive radiographic findings or signs of systemic disease. While there was a broad differential diagnosis, brain biopsy ultimately confirmed TMS.

The treatment of acute TMS flares includes high‐dose IV corticosteroids, PLEX, or IVIG [3]. High‐dose IV corticosteroids have been shown to reduce the size of tumefactive lesions [5]. PLEX has been used to treat cases of acute TMS flares refractory to corticosteroids [16]. Our patient was treated with IV methylprednisolone 1 g daily for five days during her initial presentation, with clinical improvement. The patient experienced multiple relapses, each associated with the development of new lesions. During her relapses, she received a combination of immunosuppressive treatment with high‐dose IV corticosteroids and PLEX, as illustrated in Table 1. This case highlights the treatment‐refractory course of TMS, as the patient continued to have multiple relapses with the development of new lesions.

TMS may be resistant to conventional immunosuppressive treatments and require a high‐efficacy DMT to achieve remission. In the literature, DMTs used to treat TMS include ocrelizumab, rituximab, natalizumab, and fingolimod [16–18]. Our patient initially received ocrelizumab, a monoclonal antibody targeting CD20, as a DMT. While on ocrelizumab, the patient experienced two severe, disabling relapses, suggesting that there was active inflammatory disease despite treatment. A limitation of this case is that peripheral B‐cell markers, including CD19, CD20, and CD27, were not measured while the patient received ocrelizumab. This limited our ability to assess whether B‐cell depletion had been achieved while she was on ocrelizumab. Although B‐cell markers may provide supportive objective evidence of the effectiveness of anti‐CD20 therapy on B‐cell depletion, routine B‐cell monitoring has not been incorporated into standard clinical practice [19]. Disease remission was achieved after transition to natalizumab, suggesting that natalizumab may be considered in selected severe cases, although additional evidence is needed. Natalizumab is a monoclonal antibody that targets Alpha‐4 integrin on lymphocytes, blocking their migration into the CNS [16]. While natalizumab is used to treat relapsing–remitting multiple sclerosis, there are limited case reports of natalizumab used as a DMT for TMS. Natalizumab has been associated with an increased risk of progressive multifocal leukoencephalopathy (PML), particularly among patients who are seropositive for anti‐JCV antibodies, have a history of prior immunosuppressant use, or have received prolonged treatment [20]. Given the increased risk of PML in patients treated with natalizumab, it is recommended to screen for anti‐JC virus antibodies periodically to guide risk stratification and monitoring [21].

In summary, this case details the diagnostic challenges of TMS, which may radiographically resemble infectious, neoplastic, and inflammatory conditions. In this case, brain abscess was initially strongly considered given the temporal association of the patient’s history of extensive dental caries and subsequent extractions and the development of new brain lesions. This case emphasizes the importance of early diagnostic reconsideration in atypical cases and the role of a brain biopsy in the definitive diagnosis when infectious, neoplastic, and inflammatory etiologies remain plausible. Prompt initiation of immunotherapy during acute relapses may help achieve control of active disease. TMS may cause a medically aggressive and treatment‐refractory course, requiring treatment escalation to a highly efficacious DMT for disease remission.

## Author Contributions

Pooja Patel contributed to conceptualization, investigation, data curation, visualization, writing–original draft, and writing–review and editing of the manuscript. Henrik Ehrhardt and Daniel Augh contributed to investigation, data curation, and writing–review and editing of the manuscript. Kant Matsuda contributed to investigation through review of biopsy samples and pathological confirmation of the diagnosis, as well as visualization. Kanika Sharma contributed to conceptualization, supervision, mentorship, and writing–review and editing of the manuscript.

## Funding

This project was not funded.

## Disclosure

All authors read and approved the final version of the manuscript.

## Consent

The patient provided written consent for publication.

## Conflicts of Interest

The authors declare no conflicts of interest.

## Data Availability

Data sharing is not applicable to this article as no datasets were generated or analyzed during the current study.
